# Follow-up of CRT-D patients downgraded to CRT-P at the time of generator exchange

**DOI:** 10.3389/fcvm.2023.1217523

**Published:** 2023-06-15

**Authors:** Simon Martin Frey, Roman Brenner, Dominic A. Theuns, Naeem Al-Shoaibi, Richard J. Crawley, Peter Ammann, Christian Sticherling, Michael Kühne, Stefan Osswald, Beat Schaer

**Affiliations:** ^1^ Department of Cardiology, University Hospital Basel, Basel, Switzerland; ^2^ School of Biomedical Engineering and Imaging Sciences, King’s College London, London, United Kingdom; ^3^ Department of Cardiology, Kantonsspital St. Gallen, St. Gallen, Switzerland; ^4^ Department of Cardiology, Erasmus Medical Center, Rotterdam, The Netherlands; ^5^ Department of Medicine, King Abdulaziz University, Jeddah, Saudi Arabia

**Keywords:** cardiac resynchronisation therapy (CRT), downgrade, super response, arrhythmic risk, appropriate ICD therapy, tachyarrhythmia

## Abstract

**Background:**

Some patients with cardiac resynchronisation therapy (CRT) experience super-response (LVEF improvements to ≥50%). At generator exchange (GE), downgrading (DG) from CRT-defibrillator (CRT-D) to CRT-pacemaker (CRT-P) could be an option for these patients on primary prevention ICD indication and no required ICD therapies. Long-term data on arrhythmic events in super-responders is scarce.

**Methods:**

CRT-D patients with LVEF improvement to ≥50% at GE were identified in four large centres for retrospective analysis. Mortality, significant ventricular tachyarrhythmia and appropriate ICD-therapy were determined, and patient analysis was split into two groups (downgraded to CRT-P or not).

**Results:**

Sixty-six patients (53% male, 26% coronary artery disease) on primary prevention were followed for a median of 129 months [IQR: 101–155] after implantation. 27 (41%) patients were downgraded to CRT-P at GE after a median of 68 [IQR: 58–98] months (LVEF 54% ± 4%). The other 39 (59%) continued with CRT-D therapy (LVEF 52% ± 6%). No cardiac death or significant arrhythmia occurred in the CRT-P group (median follow-up (FU) 38 months [IQR: 29–53]). Three appropriate ICD-therapies occurred in the CRT-D group [median FU 70 months (IQR: 39–97)]. Annualized event-rates after DG/GE were 1.5%/year and 1.0%/year in the CRT-D group and the whole cohort, respectively.

**Conclusions:**

No significant tachyarrhythmia were detected in the patients downgraded to CRT-P during follow-up. However, three events were observed in the CRT-D group. Whilst downgrading CRT-D patients is an option, a small residual risk for arrhythmic events remains and decisions regarding downgrade should be made on a case-by-case basis.

## Introduction

Cardiac resynchronization therapy (CRT) is an established treatment option in symptomatic patients with congestive heart failure (CHF) and left bundle branch block (LBBB), and has been shown to reduce both mortality and morbidity (1, 2). Because patients with an indication for CRT often also fulfil the indication for primary implantable cardioverter-defibrillator (ICD) implantation, most devices implanted are CRT defibrillators (CRT-D) (around 70%) (3).

A considerable subgroup of CRT patients demonstrate a “super-response”, i.e., an improvement of left ventricular ejection fraction (LVEF) to 50% or higher (1, 4–6). As different echocardiographic and clinical variables have been used in previous studies, a commonly accepted definition of super-response is not available, thus prevalence is difficult to estimate. If super-response is defined as an increase of LVEF of ≥50%, the prevalence ranges from 6% to 24% (5, 6).

Importantly, improvement of LVEF is associated with fewer arrhythmic events (1, 4, 5, 7–9). If improvement in LVEF is maintained to the first or second battery depletion, patients would not necessarily fulfil the indication for primary prevention implantable cardioverter defibrillator (ICD) at the time of generator exchange (GE). In patients with CRT-D devices, normalisation of LVEF and no previous requirement for significant ICD therapy downgrade to a CRT pacemaker (CRT-P) is becoming more common. However, in current practice less than 10% of patients are downgraded—with the most common reasons being life expectancy <1 year (61%), terminal severe heart failure (42%), and age >80 years (38%) (10). Frailty (28%) and prior inappropriate therapy (without the need for appropriate device therapy) (4%) were less frequent reasons (10).

Potential advantages of a downgrade to CRT-P include reduced risk of inappropriate ICD-shocks [∼20% (11)], decreased risk of infection (12), smaller pocket size, longer battery life and lower device costs. However, the protection against life-threatening ventricular arrhythmias is lost.

Downgrading devices with the nowadays mostly used DF-4 header requires the abandonment of the ICD lead and the additional implantation of a pace/sense lead, which complicates downgrading and involves some risks. Still, a significant number of patients with an active DF-1 system remain who need GE and in whom downgrading is a technically feasible option.

In the literature, data on long-term outcome of super-responders after GE is scarce and limited to small case series of downgraded patients (13, 14). In order to assess this further, we established a multi-centre retrospective cohort containing patients who experienced super-response by the time of GE, with a view to describing the clinical long-term outcome with respect to ventricular arrhythmias and death.

## Materials and methods

In this retrospective study patients with a CRT-D system were screened for super-response at four different centres. LVEF ≥50% at the time of GE was used to identify patients defined as super-responders. Super-responder patients were included for this study if their device was implanted for primary prevention, no arrhythmia occurred after the censor period (see below), and they had consented for use of their health care data in research.

At GE, super-responders were offered downgrade to CRT-P by the treating physician: Patients were informed that as a persistent super-responders, they would not meet the ICD indication anymore and it is unclear from current knowledge whether to continue with ICD backup or not in this situation. Advantages (no inappropriate shocks, longer battery life, smaller device) and disadvantages (small residual risk of malignant arrhythmia) were discussed and the option for downgrade offered. The final decision to downgrade was made solely by the patient according to the his/her preference. No specific clinical parameters or financial considerations influenced this decision.

Significant arrhythmia were defined as occurrence of ventricular fibrillation (VF) or sustained ventricular tachycardia (VT) at least 12 months after initial CRT-D implantation. This includes any ventricular arrhythmia requiring appropriate ICD therapy [antitachycardia pacing (ATP) and/or cardioversion/defibrillation] or sustained ventricular arrhythmia in downgraded patients. Arrythmias detected within 12 months of initial CRT-D implantation were not regarded as event since patients were thought to be still in a stage of myocardial remodelling.

Final outcomes for patients were assessed from the last recorded follow-up visit prior to closure of the database, or at the point of death or significant arrhythmia. Whenever possible, cause of death was classified as either cardiac or non-cardiac. Cardiac death was defined as severe cardiac disease in the absence of another life-limiting disease.

Information including baseline demographics, LVEF both at the time of CRT-D implantation and during follow-up, mortality, hospitalization for arrhythmias and occurrence of ICD-therapies from device interrogation were extracted from medical records and analysed retrospectively. Patients were stratified according to underlying cardiomyopathy (ischaemic heart disease (IHD) vs. non-ischaemic cardiomyopathy (NICM)). IHD was defined as heart failure judged to be ischaemic in origin due to the presence of significant epicardial stenosis. If no significant coronary artery disease was present, underlying cardiomyopathy was classified as NICM.

Data were collected at each centre and pooled for statistical analysis. All patients consented for the use of their health care data and the study was carried out according to the principles of the Declaration of Helsinki from 1975.

### Statistical analysis

Statistical analysis was performed with R (version 4.0.2) and SPSS™ (version 23). Continuous variables are expressed as mean values (± one standard deviation). Categorical variables are expressed as numbers (percentage). *T*-test and *χ*^2^ test were used where appropriate. Calculation of the cumulative event-free survival was performed with the Kaplan-Meier survival function and the Log Rank test was used to examine difference between groups. Annualized event-rate was calculated by dividing the number of events by patients-years (patients at risk multiplied by mean follow-up). No multivariable logistic regression model to explore for predictors of arrhythmic events could be performed since the event number was too low.

## Results

### Baseline characteristics

In the four participating centres, 66 patients with super-response were identified. Baseline characteristics of the patients are displayed in Table 1. First implantation of CRT-D was between 02/2000 and 04/2018. In downgraded patients, diabetes mellitus was more prevalent (33% vs. 10%, *p* = 0.05) and the QRS duration measured on 12-lead electrocardiogram (ECG) was significantly shorter (158 ms vs. 172 ms, *p* = 0.01). There were no other statistically significant differences between the groups.

**Table 1:** Baseline characteristics.

	Overall	Downgrade	Control	*p*-value
*n*=	66	27	39	
Male gender (%)	35 (53)	12 (44)	23 (59)	0.36
Age (SD)	60 (12)	63 (12)	59 (11)	0.19
EF baseline (%)	25 (7)	24 (5)	25 (8)	0.58
Non-ischaemic cardiomyopathy (%)	49 (74)	18 (67)	31 (79)	0.38
Hypertension (%)	33 (50)	14 (52)	19 (49)	1
Diabetes (%)	13 (20)	9 (33)	4 (10)	0.05
BMI (SD)	28 (6)	29 (5)	27 (6)	0.34
NYHA class (%)				0.56
II	22 (33)	7 (26)	15 (38)	
III	42 (64)	19 (70)	23 (59)	
IV	2 (3)	1 (4)	1 (3)	
Sinus rhythm (%)	60 (91)	26 (96)	34 (87)	0.41
QRS width (ms)	166 (21)	158 (21)	172 (20)	0.01
Chronic kidney disease* (%)	24 (36)	13 (48)	11 (28)	0.16
Number of generator exchanges				0.43
1 generator exchange	44 (67)	20 (74)	24 (62)	
≥2 generator exchanges	22 (33)	7 (26)	15 (38)	

Table indicating baseline characteristics at first CRT implantation. Categorical variable indicated with number of patients and percentage in brackets. Continuous variables indicated with mean and standard deviation.

* MDRD <60 ml/min.

### Follow-up

Median overall follow-up after first CRT-D implantation was 129 months (IQR: 101–155). Median follow-up after downgrade or first GE was 53 (IQR: 30–82) months. First generator exchange was performed after median 64 (IQR: 51–71) months. 21 patients (32%) underwent a second GE after median 57 (IQR: 48–70) months. At first GE, mean baseline LVEF had improved from 25% ± 7% to 53% ± 6% (*p* < 0.001). 57 (86%) patients experienced super-response at first GE. 9 patients (14%) did not achieve super-response until the second GE, although it should be noted that mean LVEF in these patients was considerably higher compared to baseline at the time of first GE (43% ± 3% vs. 26% ± 5%, *p* < 0.001). In 12 patients (18%) LVEF was assessed at the last GE. In patients with available LVEF recently before file closure, 85% remained super-responders (46/54 patients).

### Downgrades

During the follow-up period, 27 patients (41%) were downgraded from CRT-D to CRT-P. The other 39 patients (59%) had replacement CRT-D generators implanted at GE. The median time to downgrade to CRT-P was 68 months (IQR: 58–98) after implant. Mean LVEF at the time of downgrade was 54% ± 4% – this had improved significantly from baseline (24% ± 7%, *p* < 0.001). 21 patients (78% of those downgraded) were downgraded to CRT-P at first GE, whilst the other 6 patients (22%) were downgraded at second GE.

### Events during censor period

Two arrhythmia events (3%) occurred in the first 12 months following initial CRT-D implant (censor period). This period following implantation is assumed to be the time of greatest myocardial remodelling (9).

• Monomorphic VT after one month in a female patient (60 y) with non-ischemic cardiomyopathy (NICM); LVEF 26% at baseline; terminated by ATP; treatment with amiodarone was started until 8 months (stopped for intolerance);
• Electrical storm with multiple VF episodes after 6 months in a male patient (71 y) with ischemic heart disease (IHD) including apical aneurysm; LVEF 28% at baseline; further improvement in LVEF to 50% after 65 months, but deterioration later and death of congestive heart failure after 97 months.

Importantly, although the events occurred in the censor period, both cases were subsequently not considered for downgrade to CRT-P by the treating physician.

### Events after the censor period

**Figure 1:**
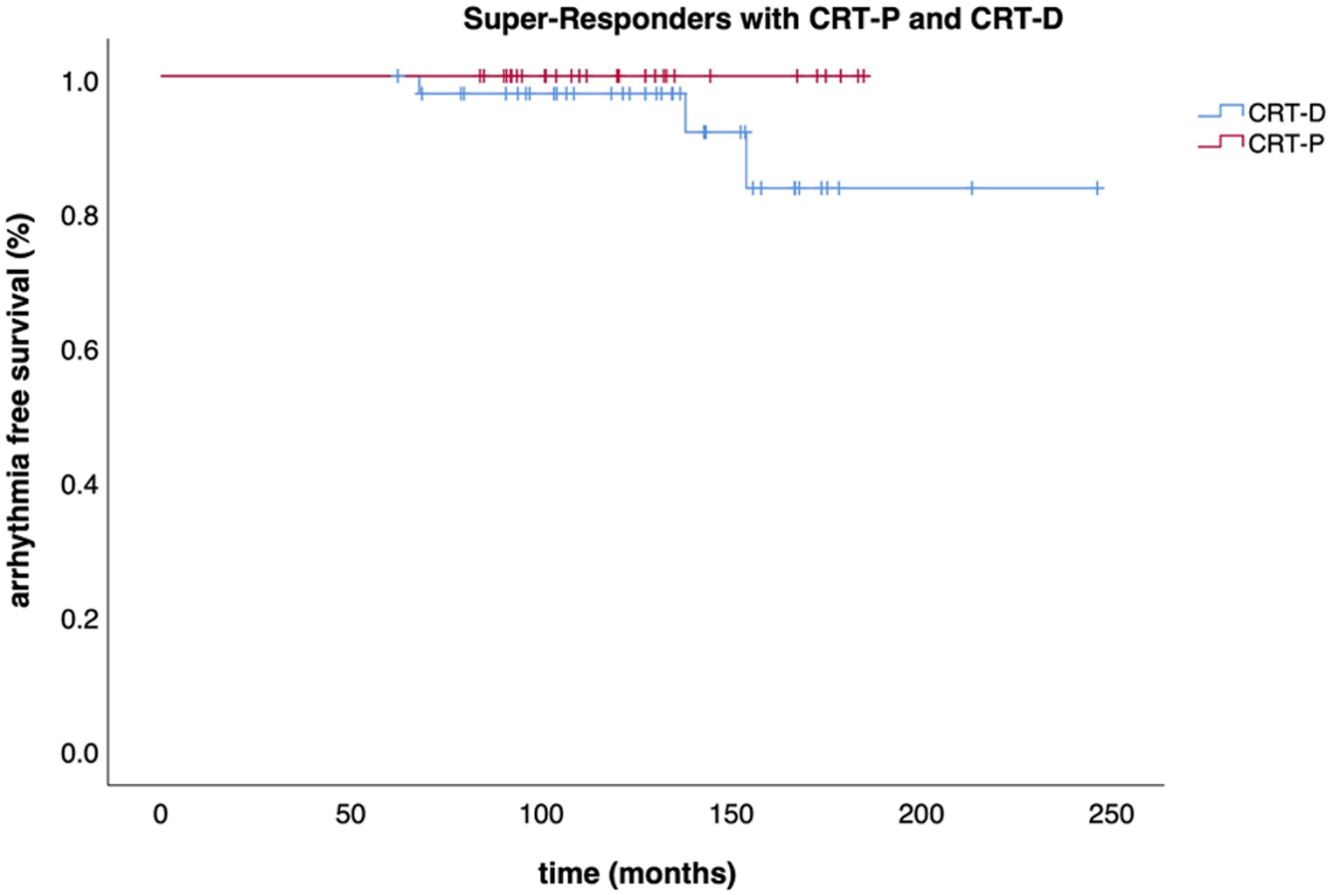
Survival free of significant arrhythmia. Kaplan-Meier curve displaying the event-free survival (significant arrhythmia) of CRT-P and CRT-D patients.

**Figure 2:**
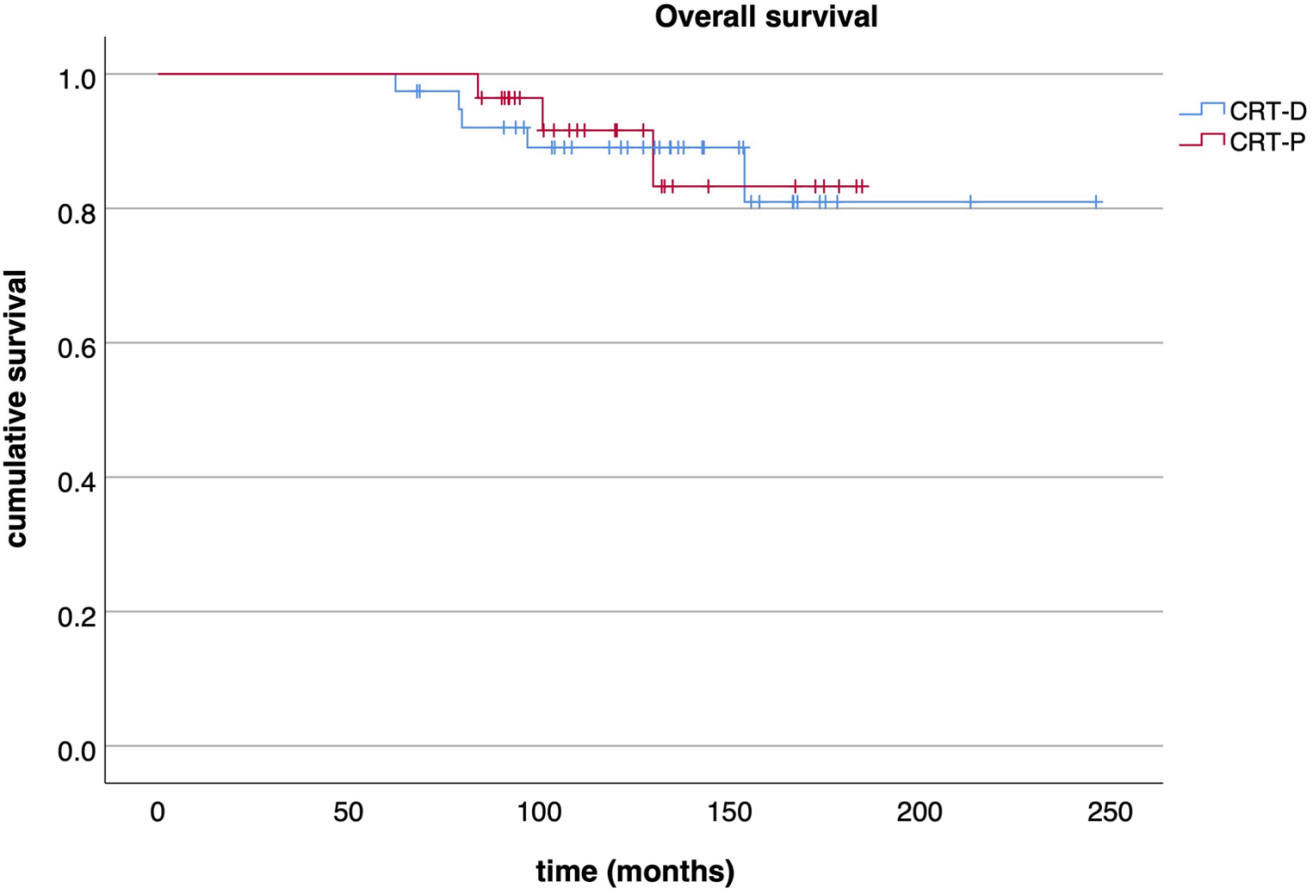
Overall survival. Kaplan-Meier curve displaying the overall survival of CRT-P and CRT-D patients.

Out of those downgraded to CRT-P, no significant arrhythmia event occurred during a median follow-up of 38 (IQR: 29–53) months after DG as assessed by device interrogation. However, three events (8%, 3/39) were observed in the patients who had CRT-D replacement after the first GE over a median follow-up of 70 (IQR: 39–97) months (Table 2). Survival free of significant arrhythmia is depicted in Figure 1. The difference in event-free survival was not statistically significant (Log Rank *p* = 0.195). There was no difference in overall survival as shown in Figure 2 (Log Rank *p* = 0.868).

**Table 2:** Arrhythmic events of three patients remaining on CRT-D despite super-response.

Patient	Cardiomyopathy	LVEF at event	Event	Therapy	Follow-up
Male 25 years	DCM (LVEF initially 20%)	55%	VT (CL 245 ms) @ 68 months triggered by sports	Several ATP 2× shock	Persistent super-response (LVEF 58%), no further arrhythmic events
Male 62 years	CAD with inferior infarction, ACBP (LVEF initially 30%)	50%	VF @ 138 months no triggering factors identified	ATP during charging	Elective PCI due to unstable angina, LVEF 54%, no further arrhythmic events
Male 38 years	DCM (LVEF initially 10%)	22% (at first GE super-response to 50%)	VT (CL 280 ms) @ 154 months	3× ATP, 4× shock	Further deterioration, LVAD implantation, progression of CHF, death due to haemorrhagic shock after 189 months

ACBP, aorta-coronary bypass; ATP, anti-tachycardia pacing; CAD, coronary artery disease; CHF, congestive heart failure; CL, cycle length; DCM, dilated cardiomyopathy; GE, generator exchange; LVAD, left ventricular assist device; LVEF, left ventricular ejection fraction; PCI, percutaneous coronary intervention; VF, ventricular fibrillation; VT, ventricular tachycardia.

Annualised event-rates after DG/GE amounted to 1.5%/year in the CRT-D group, reducing to 1.0%/year when considering the whole cohort. The annualised event-rates during total follow-up were 0.7%/year in the CRT-D group and 0.4%/year in the whole cohort.

As shown in Table 3, 8 (12%) deaths occurred after median 90 (IQR: 80–108) months, only two (25%) of these were cardiac. No cardiac death occurred in downgraded patients.

**Table 3:** Causes of death.

	Downgrade	Control
Cardiac	None	Septic + cardiogenic shock (severe pulmonary hypertension due to pneumopathy) (80 months) Congestive heart failure (97 months)
Non cardiac	Intracerebral bleeding (84 months) Neuroendocrine cancer (101 months) Stroke (130 months)	Lung disease with PH (62 months) Larynx cancer (89 months) Haemorrhagic shock (189 months)

Causes of death grouped by device status (CRT-P/CRT-D) and cardiac vs. non-cardiac death.

PH, pulmonary hypertension.

## Discussion

To the best of our knowledge, this is the largest cohort assessment of CRT-D super-responders who have been downgraded to CRT-P. With a total follow-up of more than 10 years [median 129 (IQR: 101–155) months] and more than 4 years following downgrade/first GE [median 53 (IQR: 30–82) months], the observation period is very long.

The main finding of this study is that none of the 27 super-responder patients downgraded to CRT-P suffered from an arrhythmic event or cardiac death during follow-up. This suggests that super-responders without ICD therapies may be good candidates in whom to consider downgrade of their device at generator exchange. However, this special subgroup of all CRT-D patients reflects a minority of CRT-D patients only and generalizability to all CRT-P patients is limited. But, with further improvement of heart failure treatment (“fantastic four”) and a longer follow-up of these patients, the number of super-responders will increase, and consequently the question regarding downgrade. Yet, only small cohorts of CRT-D super-responders after generator exchange have been investigated (13–16). Therefore, this study adds significant data to this important research question.

### Event rates in downgraded patients

Ogano et al. demonstrated the feasibility and mid-term safety of downgrading responders (defined as LVEF ≥45%, primary prevention, no VT/VF since implantation) (14). From this cohort of 49 consecutive patients, 7 (14%) were downgraded at GE and followed-up during an observation period of 40 ± 21 months. Similar to our cohort, downgraded patients experienced no arrhythmic events, although it should be noted that the number of downgraded patients in this study was relatively low. Additionally, the downgraded patients tended to have a lower all-cause mortality compared with those patients not deemed to be responders. This trend was not present in our cohort, which may be because all patients assessed in this study fulfilled the criteria for super-response.

Garcia and colleagues downgraded 14 patients to CRT-P following improvement of LVEF to >35% with no ICD therapies (13). Mean LVEF at GE was 49%. During a follow-up of 5.1 ± 1.3 years, there were 2 incidences of VT, but no sudden death. The research team concluded that downgrade was a safe and cost-effective treatment option. Comparability to our cohort is limited as the definitions of super-response differ significantly and the follow-up is twice as long.

### Event rates in super-responders after GE

Event rates in our study were surprisingly low for such a long follow-up period, although low event rates in super-responders were also seen in the study by House et al. (15). In 30 ICD patients with super-response (LVEF ≥ 50%), no arrhythmias were seen during a mean follow-up of 25 ± 18 months after generator exchange. However, this study has several key differences compared with our study: the follow-up period was shorter and the sample size smaller, and the cohort included 16 CRT-D patients only.

Nesti et al. observed 103 CRT-D patients for 26 ± 10 months after GE (16). Responders were defined as decrease in left ventricular end-systolic volume of ≥15%. Four responders (4% of the cohort) experienced a first arrhythmia after GE. Unfortunately, it is not reported how many of these responders who suffered from arrhythmia events were super-responders with LVEF ≥50%, which would allow direct comparison with our cohort.

### Event rates in super-responders

Arrhythmic events are rare in super-responders as shown by Ghani et al.—in this cohort of CRT-D patients followed until their first GE, none of those classified as super-responders (56/347, 16%) experienced an ICD therapy over a follow-up of 5.3 years (4).

Zecchini et al. showed that in the super-responder subgroup (24%, 62/259), only 7% had a first appropriate device therapy during a mean follow-up of 68 ± 30 months despite LVEF >50%, resulting in an annualized event rate of 1.2%/year (6). Using a similar definition of super-response, Killu and colleagues demonstrated a lower event rate of ICD therapy (0.4%/year, and a cumulative 5 year rate of 2.7%) (5). A recent meta-analysis revealed a rate of ventricular arrhythmia of 0.9%/year (17). Thus, all studies are in line with our findings (annualized event rate of 1.0%–1.5%/year after DG/GE, 0.4%–0.7%/year during total follow-up). With our study looking at a large cohort of downgraded super-responder patients over a longer follow-up period, this study adds to the growing body of evidence that downgrading to CRT-P appears safe and could be considered by clinicians.

### Predictors of super-response and survival

Certain patient characteristics are associated with super-response, such as female gender, NICM, higher LVEF at baseline, LBBB morphology, wider QRS duration, BMI <30 kg/m^2^ and smaller baseline atrial size (4, 18).

Primary prevention defibrillator was not associated with a significant lower mortality compared to a combination of CRT-P and medical therapy in symptomatic patients with NICM (DANISH-trial) (12). The majority of patients (74%) in our cohort had NICM and therefore might represent patients similar to the DANISH-trial at their first GE. As ICD was superior in the subgroups of age <59 years and NT-proBNP <1,177 pg/ml only (12), these factors should influence choice of device at GE. Furthermore, survival advantage with ICD is less clear in older (≥75 years) and diabetic patients (EU-CERT-ICD) (19). In addition, extensive myocardial scaring on cardiac magnetic resonance is a known risk factor for the occurrence of arrhythmic events (HR 5.2) (20).

Although these predictors were established at the time of initial CRT-D implantation, knowledge of these is important to guide and counsel patients at the time of GE. A clinical score usable at GE incorporating baseline and follow-up factors (e.g., age, gender, frailty, malignant arrythmia, myocardial scar, comorbidities) would be desirable. Our sample size and event rate was too small for solid assessment of such factors. But further sub-studies from larger cohorts, meta-analyses or eventually data from the RESET-CRT trial could be useful (21).

### Advantages of CRT downgrade and technical considerations

Potential advantages of a downgrade are no inappropriate ICD-shocks [which can be up to 20% (11)], lower infection rates (12) and smaller pocket size. Furthermore, the longer battery life, wider control interval and lower costs can be beneficial for certain healthcare systems. These factors gain more importance in patients with super-response and thus low risk for arrhythmic events. In a contemporary cohort study with CRT-D systems implanted after 2015, the percentage of inappropriate therapy and inappropriate shock were still 7.2% and 3.3%, respectively (22).

Different CRT-D header types exist which complicate the option of downgrading. In systems using DF-1 headers, high-voltage parts can simply be disconnected, and the pace-sense part be connected to a new CRT-P device. Despite being widely replaced by DF-4 leads, still a considerable number of patients have a functioning DF-1 lead and present themselves for GE. In patient with newer DF-4 leads, downgrade to CRT-P is not possible without implanting a new pace/sense lead and abandonment of the DF-4-ICD lead. This additional lead implantation could result in potential complications which might outweigh the benefits of a downgrade. Another possible, but manageable downside of a downgrade is that the CRT-system loses its MRI compatibility if the old ICD lead is not extracted.

### CRT in the era of the “fantastic four”

Modern pharmacological treatment markedly improved mortality and morbidity of patients suffering from heart failure (23). With the addition of two potent agents (angiotensin receptor/neprilysin inhibitors (ARNIs) and sodium–glucose co-transporter 2 (SGLT-2)) many patients nowadays have the chance to profit from these “fantastic four”. All these agents improve cardiovascular outcomes, and even reduce arrhythmia and sudden cardiac death (24–27). Furthermore, accumulating data question the mortality benefit of ICDs in primary prevention, especially in patients with NICM (12, 19, 28). Hence, it can be anticipated that the clinical scenario with super-responders presenting for device replacement will occur more often, and consequently the question regarding downgrading to CRT-P. Additionally, with rising health care costs, also economical pressure might influence this decision in the future.

### Limitations

Due to the retrospective design of this study, limitations associated with this study design are possible. Due to the lack of randomisation and a predefined protocol, this study represents real-world follow-up data with no predefined timepoints for device interrogation and echocardiography (no core lab available). But the study is based on clinical data which is also used for decision making in clinical routine.

Since not all study sites have a prospective registry, percentage of super-responders from the overall cohort could not be determined. Therefore, selection bias cannot be excluded.

The decision to downgrade was taken in consent between patient and treating physician and not in a randomised fashion. There is no data available for pharmacological heart failure treatment regarding intensity, duration and agents used. This limits the further analysis as optimal medical therapy itself was also shown to reduce mortality and prevent arrhythmia. Nevertheless, all patients were treated according to the current guidelines which includes optimal medical treatment.

To optimally analyse the debate about downgrade of super-responders, a randomized-controlled trial would be optimal. But such a study is very unlikely to ever be conducted. Hence despite the above-mentioned limitations, our study provides important long-term prognostic data on a special subgroup of CRT-patients. Since the majority of these patients had their device implanted before the novel DF-4 header was introduced, still a considerable number of these super-responder patients present with an active DF-1 lead. Knowledge of their long-term course can help clinicians in their daily decision making.

Although reverse remodelling with super-response seems to be persistent over time (29), there are still patients who can deteriorate during follow-up. In our cohort, 85% of patients remained super-responders.

## Conclusion

No super-responder downgraded to CRT-P experienced an arrhythmia event during more than 4 years of follow-up. However, 3 (8%) significant arrhythmia events occurred in those who had reimplantation of CRT-D after generator exchange leading to an annualized event-rate of 1.5%/year over subsequent follow-up. Although risk of arrhythmia is relatively low, a residual lifetime risk for sudden cardiac death remains. To validate these findings, a prospective randomised controlled trial would be needed. Meanwhile, we suggest that the decision to downgrade has to be made on a “case-by-case” basis taking into consideration factors such as age, comorbidities, scar, inappropriate ICD therapies and patient's preference.

## Data Availability

The original contributions presented in the study are included in the article, further inquiries can be directed to the corresponding author.
